# Whole-Genome Sequencing Analyses Reveal the Whip-like Tail Formation, Innate Immune Evolution, and DNA Repair Mechanisms of *Eupleurogrammus muticus*

**DOI:** 10.3390/ani14030434

**Published:** 2024-01-29

**Authors:** Fang-Yuan Han, Ren-Xie Wu, Ben-Ben Miao, Su-Fang Niu, Qing-Hua Wang, Zhen-Bang Liang

**Affiliations:** 1 College of Fisheries, Guangdong Ocean University, Zhanjiang 524088, China; hanfangyuan1999@163.com (F.-Y.H.); wolf0487@126.com (S.-F.N.); liangzhenbang0403@163.com (Z.-B.L.); 2 State Key Laboratory of Marine Environmental Science, College of Ocean and Earth Sciences, Xiamen University, Xiamen 361102, China; miaobenben@stu.xmu.edu.cn; 3 State Key Laboratory of Biocontrol, Institute of Aquatic Economic Animals and Guangdong Provincial Key Laboratory for Aquatic Economic Animals, Life Sciences School, Sun Yat-sen University, Guangzhou 510275, China; wqh31016877430@163.com

**Keywords:** *Eupleurogrammus muticus*, genome sequencing, comparative genomics, positive selection

## Abstract

**Simple Summary:**

We constructed a high-quality genome assembly of *Eupleurogrammus muticus* at the chromosomal level using PacBio SMRT, Illumina Nova-Seq, and Hi-C technologies. By combining genomic annotation, comparative genomic analyses, and species attribute information, we identified many candidate genes related to the whip-like tail, innate immunity, and DNA repair of *E. muticus*, and determined the evolutionary relationship and divergence time between *E. muticus* and related species. These findings provide important genomic resources for exploring the genetic mechanisms underlying the unique characteristics of *E. muticus* and fishery resource conservation.

**Abstract:**

Smallhead hairtail (*Eupleurogrammus muticus*) is an important marine economic fish distributed along the northern Indian Ocean and the northwest Pacific coast; however, little is known about the mechanism of its genetic evolution. This study generated the first genome assembly of *E*. *muticus* at the chromosomal level using a combination of PacBio SMRT, Illumina Nova-Seq, and Hi-C technologies. The final assembled genome size was 709.27 Mb, with a contig N50 of 25.07 Mb, GC content of 40.81%, heterozygosity rate of 1.18%, and repetitive sequence rate of 35.43%. *E*. *muticus* genome contained 21,949 protein-coding genes (97.92% of the genes were functionally annotated) and 24 chromosomes. There were 143 expansion gene families, 708 contraction gene families, and 4888 positively selected genes in the genome. Based on the comparative genomic analyses, we screened several candidate genes and pathways related to whip-like tail formation, innate immunity, and DNA repair in *E*. *muticus*. These findings preliminarily reveal some molecular evolutionary mechanisms of *E*. *muticus* at the genomic level and provide important reference genomic data for the genetic studies of other trichiurids.

## 1. Introduction

Fishes of the family Trichiuridae (Teleostei, Perciformes) are important marine fishery resources widely distributed in the tropical, subtropical, and temperate waters of the Pacific, Indian, and Atlantic Oceans [1]. There are 10 genera and approximately 45 species of trichiurids recorded worldwide [2], among which the economically capturable groups belong to the genera *Trichiurus*, *Lepturacanthus*, *Eupleurogrammus*, *Lepidopus*, and *Aphanopus* [3]. *Trichiurus*, *Lepturacanthus*, and *Eupleurogrammus* are harvested mainly from the Indo-West Pacific Oceans, while *Lepidopus* and *Aphanopus* are mainly produced from the local areas of the Atlantic Ocean [3]. There have been many reports on the fishery, resource, biology, ecology, and genetics of the genera *Trichiurus* and *Lepturacanthus* [4,5,6,7,8,9,10,11], which are commercially important fishes in the Indo-West Pacific Oceans. However, there are fewer studies on *Eupleurogrammus*, limiting the conservation and management of its biodiversity and fishery resources.

*Eupleurogrammus* consists of the smallhead hairtail (*Eupleurogrammus muticus*) and longtooth hairtail (*E*. *glossodon*), and the former has a wider distribution range and higher fishing yield. *E*. *muticus* inhabits the benthopelagic zone of the continental shelf in the northern Indian Ocean and northwest Pacific coast (depths of 30–100 m), feeding on small crustaceans, mollusks, and other fishes [3,12]. It is distributed along the coast of China, with its largest yield occurring in the northern South China Sea [13]. However, with the continuous deterioration of the global marine environment, the fragmentation of biological habitats and the long-term overfishing by humans [14,15], the yield of *E*. *muticus* on the Chinese coast has been exhibiting a significant downward trend [16]. Furthermore, the total niche width of *E*. *muticus* is smaller than that of genera *Trichiurus* and *Lepturacanthus*, and its ability to utilize resources and adapt to the environment is also weaker than that of these two genera of fish [4]. However, *E*. *muticus* population resources in the southern Yellow Sea and the northern South China Sea are still assessable and contribute to the fishing yield of trichiurids [5,17]. According to the routine fishery resource survey in the past decade, the annual catch of *E*. *muticus* accounts for about 5–10% (i.e., 15,000–30,000 t) of the total annual catch (approximately 300,000 t, 2010–2022 years) of trichiurids in the northern South China Sea.

Similar to other caudal-finless trichiurids, the body of *E*. *muticus* is elongated with no scales, laterally compressed, and ribbon-like-shaped, with well-developed sharp teeth and a tapering whip-like tail [18]. Notably, *E*. *muticus* has three unique features: (1) the adult *E*. *muticus* is relatively small, with a body length measuring 20–50 cm; (2) the body color of *E*. *muticus* is more silvery-white, with white dorsal and pectoral fins; and (3) the lateral line is not curved above the pectoral fin, showing a relatively straight line backward along the ventral margin to the caudal end [3]. Thus, the widespread distribution, resource stability, and biological traits of *E*. *muticus* suggest that it may have a unique genomic characterization. However, only a few studies have investigated the biology of *E*. *muticus* in the northern Indian Ocean [12,19,20,21] and its genetic variation in the Yellow Sea of China [16,22]. There is also a lack of research to reveal the genetic and evolutionary mechanisms of the species at the genomic level. Although the genome of *Lepturacanthus savala*, belonging to the family Trichiuridae, and several genes and pathways related to its specific morphological and behavioral characteristics have been established [23], the exact molecular mechanisms associated with these features are not yet clear. Therefore, there is an urgent need to further explore the genomic information of other trichiurids with unique species attributes, such as *E*. *muticus*, to gain a comprehensive and in-depth understanding of the molecular mechanisms underlying the formation of this particular taxon of trichiurids.

In this study, we combined the PacBio SMRT, Illumina Nova-Seq, and Hi-C technologies to obtain high-quality chromosome-level genomic data for *E*. *muticus*. The molecular mechanisms associated with the whip-like tail, innate immunity, and DNA repair of *E*. *muticus* were further explored based on genome annotation, gene family contraction and expansion analyses, positive selection analysis, and species attributes. The phylogenetic relationship and divergence time between *E*. *muticus* and other fishes were also evaluated using the constructed genome phylogenetic tree. The results of this study provide a solid foundation for understanding the genetic composition, evolutionary history, and ecological adaptations of *E*. *muticus* and accurate reference genomic data for the genetic resources of other trichiurids.

## 2. Materials and Methods

### 2.1. Fish Capture and Sampling

Two live female *E*. *muticus* (specimen numbers 20210826015 and 20210826016) were caught on 30 September 2021 during a bottom trawl survey of inshore fishery resources in Wuchuan City, Guangdong Province, China. The full body length, preanal length, and body weight of specimen 20210826015 were 38.5 cm, 9.8 cm, and 39.5 g, respectively, while those of specimen 20210826016 were 38.2 cm, 9.8 cm, and 42.3 g, respectively. These two fish were anesthetized with MS-222 (ethyl 3-aminobenzoate methanesulfonate, Sigma-Aldrich, Shanghai, China) at a concentration of 200 mg/L and immediately dissected for sterile anatomical sampling. The muscle, liver, brain, and heart tissues of each fish were collected separately in 1.5 mL sterile tubes and quickly frozen in liquid nitrogen. The frozen samples and fish bodies were transferred to a −80 °C refrigerator at the Guangdong Ocean University for storage.

### 2.2. DNA and RNA Extraction and Sequencing

**Figure 1:**
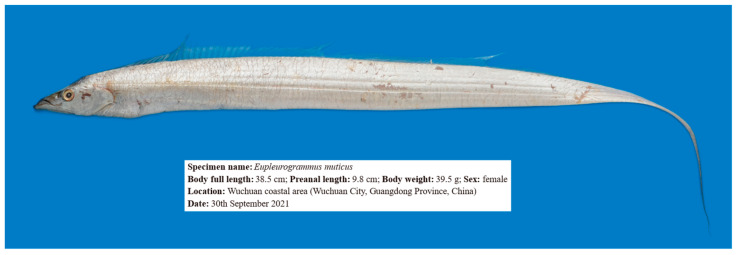
*E*. *muticus* used for sequencing.

After extracting DNA from all tissues of each fish and conducting the quality inspection, one *E. muticus* (20210826015, Figure 1) with good DNA quality tissue samples was selected for subsequent genome sequencing. The genomic DNA from muscle tissue was extracted using the standard phenol/chloroform extraction protocol [24], and the DNA concentration, purity, and integrity were detected using Qubit 3.0 Fluorometer (Invitrogen, Carlsbad, CA, USA), Nanodrop 2000 Spectrophotometer (Thermo Fisher Scientific, Waltham, MA, USA), and Agilent 4200 Bioanalyzer (Agilent Technologies, Palo Alto, CA, USA), respectively. Subsequently, a paired-end sequencing library with an insertion length of 350 bp was constructed using the Illumina TruSeq Nano DNA Library Prep Kit (Illumina, San Diego, CA, USA) and sequenced on the Illumina NovaSeq-6000 platform (Illumina, San Diego, CA, USA). Raw reads from the Illumina sequencing were quality-filtered by FASTQ v0.23.2 [25], according to the setting criteria. Furthermore, SMRTBell template preparation kit 1.0 (Pacific Biosciences, Menlo Park, CA, USA) was used to construct the SMRTbell library with a fragment size of 20 kb from the same genomic DNA used for Illumina sequencing, following the manufacturer’s protocol. After library construction, the accurate quantification and size of the SMRTbell library were detected using the Qubit 3.0 Fluorometer and Agilent 2100 Bioanalyzer (Agilent Technologies, Palo Alto, CA, USA), respectively. The library was then sequenced on the PacBio Sequel II platform (Pacific Biosciences, Menlo Park, CA, USA).

The total RNA from the muscle, liver, brain, and heart tissues was sequenced to assist in the annotation of the *E. muticus* genome. RNA was extracted using TRIzol Reagent (Invitrogen, Carlsbad, CA, USA), and its concentration and integrity were checked by Agilent 4200 TapeStation (Agilent Technologies, Palo Alto, CA, USA) and Agilent RNA ScreenTape Assay (Agilent Technologies, Palo Alto, CA, USA), respectively. Thereafter, the RNA samples from the four tissues were evenly mixed for RNA library construction and sequencing. The mRNA was then purified from the mixed RNA using magnetic beads with Oligo (dT). The purified mRNA was reverse transcribed using Reagent TUREscript First Stand cDNA Synthesis Kit (AidLab, Beijing, China) to synthesize a double-stranded cDNA library, and the Illumina pair-end sequencing library with an insert of about 350 bp was constructed. PCR Barcoding Kit (SQK-PBK004, Oxford Nanopore Technologies, Oxford, UK) and PCR-cDNA Sequencing Kit (SQK-PCS109, Oxford Nanopore Technologies, Oxford, UK) were used to construct a nanopore full-length transcriptome library. The Illumina and Nanopore libraries were then sequenced on the Illumina NovaSeq-6000 and PromethION sequencer (Oxford Nanopore Technologies, Oxford, UK) platforms, respectively.

### 2.3. Evaluation of Genome Contamination, Size, Heterozygosity, and Repeat Sequence Rate

The Illumina sequencing raw reads of muscle DNA were filtered using FASTQ v0.23.2 to obtain clean reads for the preliminary estimation of genomic features. Blastn v2.11.0+ [26] was used to map 10,000 randomly selected clean reads (5000 for Read1 and 5000 for Read2) to the NCBI nucleotide database and rank the mapping times in descending order to show the top 80% of the mapped species. If all the mapped results were homologous, it was considered that there was no exogenous pollution in the sample. Genome size and heterozygosity were estimated using the GCE v1.0.0 [27] based on the K-mer frequency distribution method. Genome size (unit: Megabits) was calculated as the number of K-mer/depth of K-mer (K-mer = 17). The depth of K-mer was the expected value corresponding to the Poisson distribution. The corrected genome size was obtained after eliminating the error effect caused by the wrong K-mer. The heterozygosity rate is the estimated proportion of heterozygous sites in the sequence. The repeat sequence rate was calculated based on the area difference between the standard Poisson distribution and the actual data curve after the peak.

### 2.4. Genome Assembly and Assessment of Genomic Integrity and Consistency

The raw sequencing data obtained via PacBio SMRT technology contained a dumbbell-shaped structural sequence of two adaptors, called polymerase reads. Subreads were obtained after the adaptor sequences were interrupted and removed, and then the high-precision HiFi reads were generated via the Circular Consensus Sequencing (CCS) mode using SMRT-Link v10.2 [28]. The HiFi reads were then assembled using Hifiasm v0.16.1 [29], and the assembled genome was de-redundantly processed by Purge Haplotigs v1.0.4 [30].

Several methods were applied to evaluate the integrity and consistency of genome assemblies. First, the base composition and content of the genome sequence were statistically analyzed to preliminarily assess the assembly results. Secondly, the genomic sequences were interrupted in steps of 1000 bp and then compared against the NCBI nucleotide database using Blastn v2.11.0+, revealing the top five genera of the comparison results to confirm whether the assembled genome belongs to the target species. Thirdly, Illumina clean reads and PacBio HiFi reads were mapped to the genomic sequences using BWA v0.7.12 [31] and Minimap2 v2.22 [32], respectively. The integrity of the genome assembly and the uniformity of sequencing were assessed based on the mapping rate, coverage rate, and sequencing depth. Fourthly, mutations in the assembled genome were identified using Samtools v1.9 [33], Picard v1.124, and GATK v4.2.0.0, and the homozygous and heterozygous rates of single nucleotide polymorphisms (SNPs) and insertion-deletion (InDel) were counted, respectively. Finally, tblastn v2.11.0+ [26], Augustus v3.3.2 [34], and HMMER v3.3.1 [35] were used to map the assembled genome sequences to the single-copy orthologous gene database based on the Benchmarking Universal Single-copy Orthologs (BUSCO) evaluation method [31], and the genome integrity was evaluated according to the mapping results.

### 2.5. Genome Assembly at the Chromosomal Level

To obtain a high-quality genome, we further applied the Hi-C technology for genome assembly at the chromosomal level. First, the muscle tissue cells were treated with 40 mL of 2% formaldehyde solution (Sbjbio, Nanjing, China) for DNA cross-linking, and the sticky ends were generated by restriction endonuclease cleavage. Secondly, biotin-labeled oligonucleotide ends were added during end repair, and adjacent DNA fragments were ligated using nucleic acid ligase. Finally, the cross-linked protein and DNA were released, and biotin-labeled DNA fragments were captured to construct the Hi-C library, which was then sequenced on the Illumina NovaSeq-6000 platform.

The raw Hi-C sequencing data were filtered via standard quality control to obtain clean data, which included multiple types of reads such as valid pairs, contiguous sequences, internal fragments, and PCR repeats, among which only the valid pairs reflect the information of site-to-site interactions on the genome [36]. Therefore, the clean data were further filtered by the hicup_ filter subroutine to obtain valid pairs, which were used as Di-Tags. The contigs or scaffolds of the same chromosome can be sorted and oriented based on the following criteria: cis interactions are much larger than trans interactions, and the closer the linear distance in cis interactions, the stronger the interactions [36]. Accordingly, the 3D-DNA program [37] was used to assemble Di-Tags and to cluster the assembled contig and scaffold sequences to obtain a chromosome-level genome. The interaction map of the assembled genome was constructed using Juicer v1.6 [38] and visualized via JuiceBox v1.11.08 [39].

### 2.6. Genome Prediction and Annotation

Genome annotation mainly includes repetitive sequence annotation, gene annotation (prediction of gene structure and function), and non-coding RNA (ncRNA) annotation. Repetitive sequences consist of tandem and interspersed repeats. Tandem repeats in the genome sequence were searched by TRF v4.09 [40], while the interspersed repeats were identified via homology prediction and de novo prediction methods. The homology prediction was based on the homologous repeat database Repbase [41]. RepeatMasker v4.0.9 and RepeatProteinMask v4.0.9 [42] were used to identify sequences with similar repeat sequences of known nucleic acids and amino acids, respectively. The de novo prediction was achieved through the RepeatMasker program based on the creation of a new repeat sequence database using LTR_Finder v1.0.7 [43] and RepeatModeler v1.0.11 [44].

The location and structure of the protein-coding genes were predicted using three methods: homology-based, de novo, and transcriptome-based prediction. The homology-based prediction involved first downloading the genome-wide protein sequences of *Homo sapiens*, *Danio rerio*, *Thunnus maccoyii*, *Thunnus albacares*, *Takifugu rubripes*, *Oryzias latipes*, *Etheostoma spectabile*, *Sander lucioperca*, *Perca flavescens*, and *Larimichthys crocea* from the NCBI database and aligning them to the *E. muticus* genome using tblastn (E-value ≤ 1 × 10^−5^). The alignments were then analyzed using GeneWise v2.4.1 [45] to define the protein-coding gene models. De novo prediction was performed via Augustus v3.3.2 and GeneScan v1.0.0 [46]. The RNA-Seq data of the tissues were assembled by Tophat v2.1.1 for comparison and Cufflinks v2.2.1 [47] to obtain the transcripts. Subsequently, the gene sets predicted by these methods were integrated into a non-redundant and more complete gene set through Maker2 v2.31.10 [48], and the results of the Core Eukaryotic Genes Mapping Approach (CEGMA) [49] were integrated using the HiCESAP process to obtain the final reliable gene set. Finally, the tblastn program was used to map the gene sequences to non-redundant (NR), Swiss Protein institute (SwissProt) [50], Kyoto Encyclopedia of Genes and Genomes (KEGG) [51], and gene ontology (GO) [52] databases. PFam [53] and InterPro databases were then used to predict the protein family and conserved functional domains of protein-coding genes.

The transfer RNA (tRNA) sequences in the genome can be searched by tRNAscan-SE v1.3.1 [54] based on their structural characteristics. Since ribosomal RNAs (rRNAs) are highly conserved, the rRNA sequences of the species closely related to *E. muticus* were selected as reference sequences, and blastn alignment was used to find rRNAs in the genome. MicroRNA (miRNA) and small nuclear RNA (snRNA) sequences in the genome were predicted by infernal software of Rfam based on the covariance model of the Rfam v14.0 family [55].

### 2.7. Gene Family Identification and Dynamics Analysis and Phylogenetic Tree Construction

Genomic comparative analysis was conducted between *E*. *muticus* and 19 other selected fishes (*L. savala*, *T. albacares*, *T. maccoyii*, *Epinephelus akaara*, *Epinephelus fuscoguttatus*, *P. flavescens*, *L. crocea*, *S. lucioperca*, *Acanthopagrus latus*, *Cheilinus undulatus*, *Echeneis naucrates*, *Periophthalmus magnuspinnatus*, *T. rubripes*, *Takifugu flavidus*, *Tetraodon nigroviridis*, *Monopterus albus*, *Hippocampus comes*, *Latimeria chalumnae*, and *Rhincodon typus*) to identify the gene family of *E*. *muticus*. First, the protein-coding genes with less than 30 amino acids were excluded from the genomes of all species, retaining the protein sequence of the longest transcript. Secondly, the similarity relationship among the protein sequences of all species was calculated by all-vs-all blastp v2.11.0+ (E-value = 1 × 10^−5^). Finally, orthologous genes were clustered by OrthoMCL v2.0.9 [56] (expansion coefficient = 1.5) based on their similarity to obtain single-copy genes and gene families.

All single-copy genes were aligned with multiple sequences using MAFFT v7.487 [57], and the results were combined into a super alignment matrix. The maximum likelihood (ML) phylogenetic tree of the above 20 species was constructed using RAxML v8.2.12 [58]. Based on the constructed phylogenetic tree, seven divergence times were obtained from the TimeTree database [59] to be used for calibration, including *R. typus* and *L. chalumnae* (442.7–515.5 Mya), *L. chalumnae* and *P. magnuspinnatus* (424.2–440 Mya), *E. muticus* and *T. albacares* (30.1–58.1 Mya), *M. albus* and *P. flavescens* (103.7–176.0 Mya), *A. latus* and *T. rubripes* (59.3–142.9 Mya), *C. undulatus* and *L. crocea* (63.9–114.6 Mya), and *T. maccoyii* and *M. albus* (106–144 Mya). Interspecific divergence times (95% confidence interval) of the above 20 species were estimated according to the seven divergence times using the default parameters of McMcTree v4.9 [60] in the PAML package [61]. Furthermore, the expansion and contraction analyses of the gene family were conducted using CAFE5 v5.0.0 [62], and the GO and KEGG enrichment analyses were further performed to explore the function of the genes and the biological processes involved. GO terms or KEGG pathways with a *p*-value < 0.05 and an FDR (false discovery rate) < 0.05 were considered to be statistically significant.

### 2.8. Positive Selection and Collinearity Analyses

Three groups of positive selection analyses were set up to screen candidate genes related to the unique traits of *E. muticus*. Group 1 (*E. muticus* and *L. savala*) vs. (*A. latus*, *E. fuscoguttatus*, *E. akaara*, *P. flavescens*, *L. crocea*, and *S. lucioperca*) and group 2 (*E. muticus*) vs. (*A. latus*, *E. fuscoguttatus*, *E. akaara*, *P. flavescens*, *L. crocea*, and *S. lucioperca*) were set to screen for genes associated with the body characteristics of *E. muticus*. Group 3 (*E. muticus* and *L. savala*) vs. (*T. albacares* and *T. maccoyii*) was set to screen the positively selected genes between the trichiurids and closely related species. The target species were identified as the foreground branch, and the remaining species as background branches. The positive selection effects acting on protein-coding sequences were detected by CodeML v4.9 [60] in PAML based on the branch-site model. The protein sequences in each single-copy gene were subjected to multiple alignments using MAFFT v7.487, and the result was subjected to a multiple-sequence alignment of the coding sequences. The likelihood values were calculated using the branch-site model analysis [63] based on two models (Model A and null mode), and the values were further subjected to likelihood ratio tests (LRTs) via the chi2 program in PAML (with a correct *p*-value of FDR < 0.05). The posterior probability of the positive selection was calculated using the Bayes empirical Bayes method (BEB) [64]. Finally, GO and KEGG enrichment analyses were performed on the positively selected genes to explore their gene functions.

There are two types of collinearity analysis: coding sequences collinearity (at the protein level) and genome-wide collinearity (at the DNA level) [65]. These two types of collinearity analyses were performed between *E. muticus* and *L. savala* using JCVI v1.1.22 [66] (for coding sequence collinearity) and Mummer v4.0.0rc1 [67] (for genome-wide collinearity).

## 3. Results

### 3.1. Genome Sequencing Data

A total of 475,992,654 raw paired reads (71.4 Gb) were generated by Illumina sequencing, and 421,436,106 clean paired reads (63.03 Gb) were obtained after data filtering and de-redundancy (Table 1). The GC content of the clean reads was 39.92%, and the proportions of base quality at >Q20 and >Q30 were 97.04% and 92.23%, respectively. The mapping results showed that the top 80% of the 29 genera were all Actinopterygii, with Perciformes having the most genera (21 genera, Table S1), including *Epinephelus* (9.58%), *Lateolabrax* (8.23), *Plectropomus* (6.57%), *Trachurus* (4.78%), *Sparus* (4.55%), *Larimichthys* (4.55%), and *Nibea* (4.18%), among others. This indicated that the Illumina sequencing data were reliable and not contaminated by external sources. The depth of K-mer was determined to be 79 according to the expected value of the Poisson distribution with K-mer = 17 (Figure S1). Based on this, the estimated genome size of *E. muticus* was 673 Mb, which was revised to 664 Mb, with a genomic heterozygosity rate of 1.18% and a repeat sequence rate of 35.43%.

### 3.2. Genome Assembly and Evaluation

In total, 2,337,277 high-quality HiFi reads (34.97 Gb) were obtained by PacBio SMRT sequencing (Table 1). The average length of the HiFi reads was 14,963.26 bp, and their N50 length was 15,643 bp. After assembly error correction and elimination of redundant sequences, 156 contigs were obtained, with the maximum (max) length, N50 length, and N90 length of 50,599,264 bp, 25,347,879 bp, and 4,252,074 bp, respectively (Table S2). The genome size assembled at the contig level was 709.27 Mb, which is close to the estimated value (664 Mb) of the genome survey.

The mapping rate of Illumina clean reads was 99.50%, with an average sequencing depth of 86.61× and a genome coverage rate of 99.93%. However, the mapping rate, average sequencing depth, and genome coverage rate of PicBio HiFi reads were 99.99%, 48.52×, and 99.99%, respectively. The correlation graph between the GC content and average depth distribution showed that the GC content was concentrated around 40.81% (Figure S2), without significant GC content separation, indicating that there was no exogenous pollution in the genome. SNP and InDel identification analyses showed that the homozygous SNP and InDel rates were 0.001% and 0.002%, and the heterozygous SNP and InDel rates were 0.936% and 0.294%, respectively. The extremely low rate of homology SNPs indicated that the assembled genome had a high single-base accuracy.

Based on the BUSCO method, the database with 3640 orthologous single-copy genes was used as a reference to evaluate the integrity of the assembled genome. The results showed that the *E. muticus* genome contained 3534 (97.1%) complete BUSCOs, of which 3471 (95.4%) were complete single-copy BUSCOs, 63 (1.7%) were complete duplicated BUSCOs, 12 (0.3%) were fragmented BUSCOs, and 94 (2.6%) were missing BUSCOs (Table S3). This suggested that the assembled genome contained over 97.1% of orthologous genes, indicating a high gene coverage rate.

### 3.3. Hi-C Technology-Assisted Genome Assembly at the Chromosomal Level

A total of 501,341,846 raw paired reads (75.20 Gb) were obtained by Illumina sequencing (Table 1), and 495,369,140 clean reads (74.25 Gb) were obtained after quality control, with 96.54% of Q20 and 91.06% of Q30. The mapping results exhibited that 132,890,440 read1 and 132,890,440 read2 were successfully matched (Table S4). Among them, valid pairs accounted for 70.62% (93,847,523), and the proportion was 51.23% (68,084,802) after de-duplication (Table S5, Figure S3).

**Figure 2:**
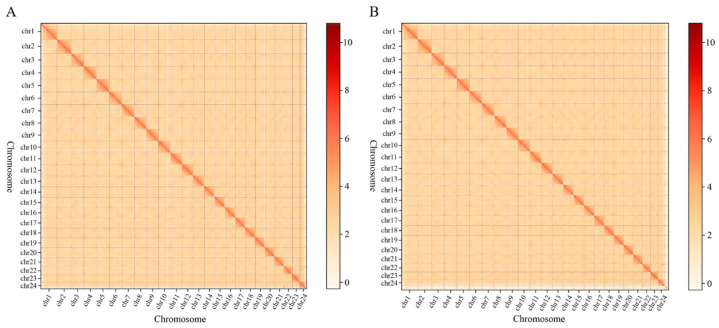
Chromosome interaction mapping (**A**) and genome-wide interaction mapping (**B**). The color reflects the intensity of each contact, with deeper colors representing higher intensity.

The Hi-C-assembled genome at the chromosomal level had 84 contigs (N50 length: 25,078,085 bp) and 60 scaffolds (N50 length: 30,064,390 bp). Among these, 24 sequences (691,679,068 bp) were assembled, while 60 sequences (10,817,913 bp) were not assembled at the chromosomal level, resulting in a genome assembly mounting rate of 98.46% (Table S6). As shown in Figure 2, the species chromosome interaction mapping was consistent with the genome-wide interaction mapping, indicating that the Hi-C-assisted assembly was good.

### 3.4. Genome Annotation

In total, 72,738,949 bp tandem repeats and 216,702,465 bp interspersed repeats were predicted, accounting for approximately 10.35% and 30.85% of the genome, respectively. Transposable elements (TEs) accounted for the highest number of repeats (102,347,937 bp; 14.57%), followed by long interspersed nuclear elements (LINEs) (36,949,851 bp; 5.26%), long terminal repeats (LTRs) (25,110,984 bp; 3.57%), and short interspersed nuclear elements (SINEs) (9,190,875 bp; 1.31%) (Table S7). A total of 29,052 non-coding RNAs were predicted in the genome, including 879 miRNAs (75,630 bp; 0.0108%), 12,792 tRNAs (968,630 bp; 0.1379%), 13,817 rRNAs (1,993,777 bp; 0.2838%), and 1537 snRNAs (236,834 bp; 0.0337%) (Table S8).

**Figure 3:**
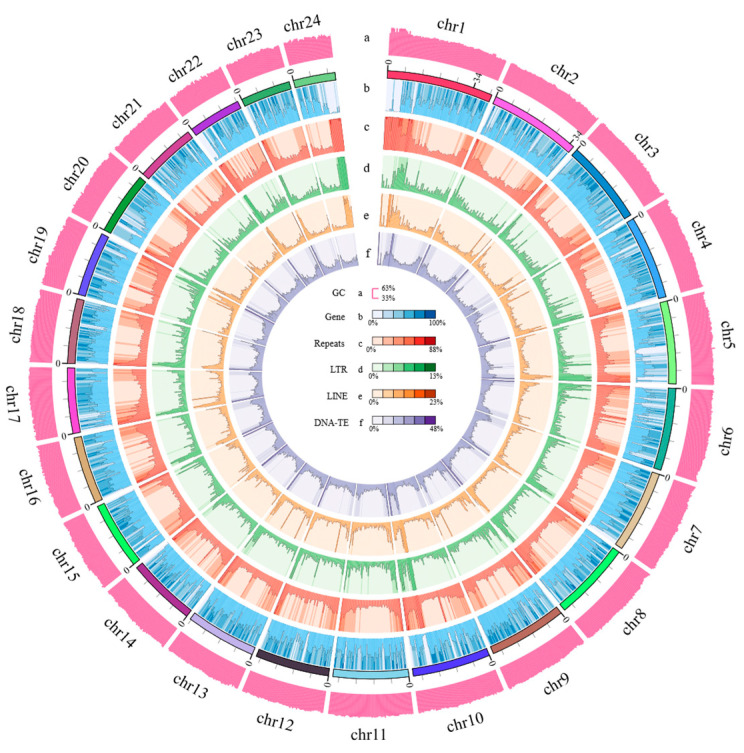
Circle figure of the genomic characteristics of *E*. *muticus*, including (a) the GC content of the genome, (b) the distribution of genes, (c) the distribution of repeats, (d) the distribution of long tandem repeats, (e) the distribution of long interspersed nuclear elements, and (f) the distribution of DNA transposable elements.

As illustrated in Figure 3, the circle figure of the genome characteristics of *E*. *muticus* was constructed based on the 24 assembled chromosomes, which showed the distribution of protein-coding genes, repeats, LTR, LINE, and DNA-TE on the 24 chromosomes. Among them, there were 21,492 genes with functional annotation, accounting for 97.92% of the total protein-coding genes (21,949) (Table S9). The basic details of 21,446 genes were obtained from the NR database, while those of 19,425 genes were obtained from the SwissProt databases. Moreover, the biological processes and functions of 15,253 genes were obtained from the GO database, while those of 21,322 genes were derived from the KEGG databases. Annotation information on the functional domains and protein families of 19,218 genes was acquired from the Pfam databases, while that of 19,940 genes was obtained from InterPro databases. The average lengths of all genes and coding sequences (CDS) were 15,160 bp and 1728 bp, respectively. The average number of exons per gene was 10.27, and the average lengths of exons and introns were 259.53 bp and 1348 bp, respectively (Table 2). The gene structure comparison between *E. muticus* and the other 10 fishes showed that the distribution of exon and CDS lengths was highly consistent among all fishes (Figure S4), indicating their conservation during fish genome evolution.

### 3.5. Gene Family Clustering, Expansion, and Contraction and Phylogenetic Analyses

**Figure 4:**
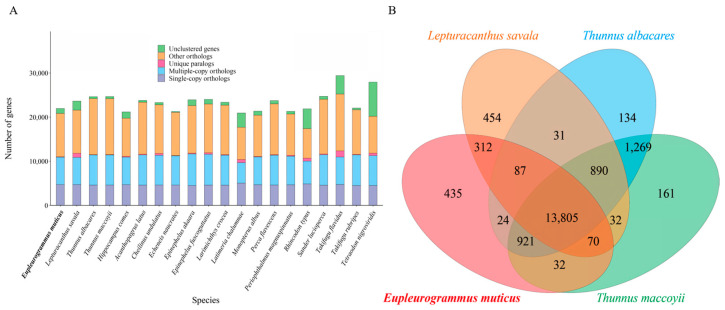
The numbers of homologous genes in 20 fish species (**A**) and Venn diagram of the homologous gene families between *E. muticus* and three closely related species (**B**).

The protein-coding genes screened from the genomes of *E. muticus* and 19 other fish species ranged from 20,932 (*L. chalumnae*) to 29,408 (*T. flavidus*) (Figure 4A), and the cluster analysis yielded 3006 single-copy genes (Table 3). In the *E.muticus* genome, 20,826 genes were clustered into 15,686 gene families, of which 6711 were common families and 25 were unique. There were 13,805 gene families shared by *E*. *muticus* and 3 closely related species (*L*. *savala*, *T*. *albacares*, and *T*. *maccoyii*), while 435 gene families were unique to *E. muticus* (Figure 4B). KEGG enrichment analysis showed that these unique gene families were mainly involved in several pathways, such as neutrophil extracellular trap formation, systemic lupus erythematosus, alcoholism, shigellosis, transcriptional misregulation in cancer, the cytosolic DNA-sensing pathway, and herpes simplex virus 1 infection (Supplementary Material S2).

**Figure 5:**
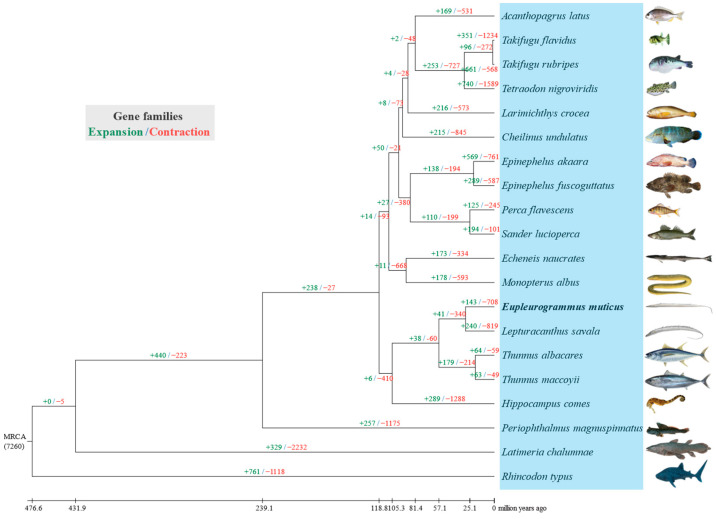
The expanded and contracted gene families of the 20 fish species during in the evolutionary process.

Based on the comparison of common ancestors between *E. muticus* and *L. savala*, 143 gene families expanded while 708 gene families contracted during the genomic evolution of *E. muticus* (Figure 5). Additional GO and KEGG enrichment analyses revealed 31 significantly expanded gene families (including 124 genes) and 123 significantly contracted gene families (including 52 genes) (Supplementary Material S3). KEGG enrichment analysis also revealed that the expanded gene families were involved in the following pathways: cytokine–cytokine receptor interaction, viral protein interaction with cytokine and cytokine receptor, chemical carcinogenesis-DNA adducts, Notch signaling pathway, and breast cancer; however, the contracted gene families were mainly involved in ABC transporters, axon guidance, antifolate resistance, antigen processing and presentation, and longevity regulating pathway-multiple pathways (Table 4). According to GO enrichment analysis, the expanded gene families were mainly enriched in various terms, including cellular processes, binding, metabolic processes, cellular metabolic processes, nitrogen compound metabolic processes, primary metabolic processes, and organic substance metabolic processes; however, the contracted gene families were mainly enriched in binding, organic cyclic compound binding, heterocyclic compound binding, catalytic activity, cellular process, ATP binding, and adenyl nucleotide binding. In general, the expanded gene families were mainly concentrated in metabolic processes, while the contracted families were mainly associated with binding processes.

**Figure 6:**
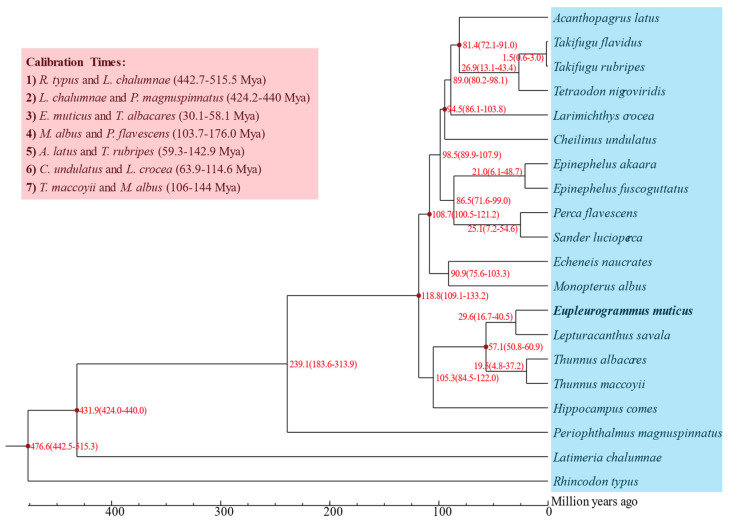
Divergence time estimates of the 20 fish species.

The ML phylogenetic tree was constructed based on the sequences of 3006 single-copy genes shared by 20 fish species (Figure S5). The results showed that *E. muticus* first clustered together with *L. savala* and then formed a sister–group relationship with *T*. *albacares* and *T*. *maccoyii*, and the nodes of all branches had 100% bootstrap support. Based on the estimated divergence time (Figure 6), the divergence between *E. muticus* and *L. savala* occurred 29.6 (16.7–40.5) million years ago, and they differentiated with *T*. *albacares* and *T*. *maccoyii* approximately 57.1 (50.8–60.9) million years ago.

### 3.6. Positive Selection and Collinearity Analyses

**Figure 7:**
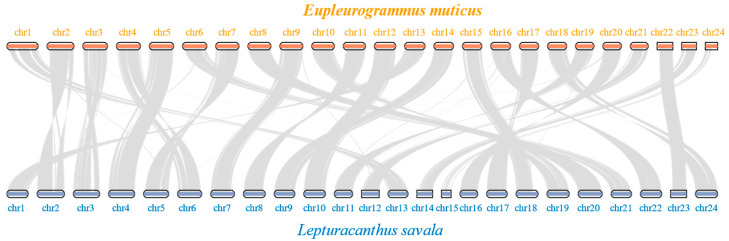
Collinearity analysis of *E. muticus* and *L. savala* based on coding sequences.

The results of positive selection analyses (Table 5, Supplementary Material S4) showed that there were 1566 positively selected genes and 21 significantly enriched pathways (mainly Fanconi anemia, non-homologous end-joining, homologous recombination, ferroptosis, and cytokine–cytokine receptor interaction pathways) detected in group 1. In group 2, there were 1022 positively selected genes and 20 significantly enriched pathways (mainly cytokine–cytokine receptor interaction, lysosome, JAK-STAT signaling pathway, Fanconi anemia, and homologous recombination). There were 2300 positively selected genes and 17 significantly enriched pathways (mainly cytokine–cytokine receptor interaction, viral protein interaction with cytokine and cytokine receptor, base excision repair, Fanconi anemia, and complement and coagulation cascades) in group 3. As shown in the two collinearity analysis figures (Figure 7 and Figure S6), the genomes of *E. muticus* and *L. savala* had a high degree of collinearity, with a one-to-one correspondence of the 24 chromosomes of the two species.

We further analyzed the functions of the positively selected genes and gene families by combining the biological characteristics, geographic distribution, and habitat environmental features of *E*. *muticus*, and confirmed that two gene families (*ccr3* and *ccr5*), 29 genes (*atg5*, *atg7*, *map1lc3b*, *map1lc3c*, *ids*, *lipa*, *gla*, *man2b1*, *glns*, *dnase2*, *ppt1*, *lamp2*, *il1rl1*, *il17a*, *c1s*, *c1qa*, *c9*, *mif*, *fanca*, *fance*, *fanci*, *faap100*, *eme1*, *brip1*, *blm*, *slx1a*, *polh*, *palb2*, and *brca1*), and six pathways (autophagosome, lysosome, cytokine–cytokine receptor interaction, complement and coagulation cascades, virus protein interaction with cytokine and cytokine receptor, and Fanconi anemia pathway) play important roles in the whip-like tail formation, innate immune evolution, and DNA repair mechanisms of *E*. *muticus*.

## 4. Discussion

### 4.1. Characterization of the E. muticus Genome

In this study, we combined PacBio SMRT-Seq, Illumina Nova-Seq, and Hi-C technologies to obtain the first chromosome-level genome assembly of *E*. *muticus*. Since high coverage is one of the necessary conditions for sequencing error correction, genome coverage is a key indicator for measuring the efficiency of high-throughput genome sequencing technology [68]. Contig N50 value reflects the size and potential continuity of the genome assembly and is an important parameter for determining the quality of genome assembly [69]. In this study, 222.36 Gb of raw sequencing data were obtained from different sequencing technologies, with Q20 and Q30 values above 95% and 90%, respectively, and a genome coverage of 274.4×. The contig N50 generated by the assembly was 25.07 Mb, and the proportion of genes with a complete comparison to BUSCO reached 97.1%. These high coverage and large contig N50 values showed the accuracy of the sequencing data and the high quality of the assembled *E*. *muticus* genome. The size of the final assembled genome was 709.27 Mb, with a GC content of 40.81%, a heterozygosity rate of 1.18%, and a repetitive sequence rate of 35.43%. These values were generally close to the results of the *E*. *muticus* genome survey (670 Mb, 41.68%, 1.26%, 35.33%) reported by Song et al. [70], but differed significantly from the results of the *L*. *savala* genome (790.02 Mb, 39.03%, 0.53%, 40.54%) as determined by Wu et al. [23]. The rate of repetitive sequences is a key factor influencing the genome size of species [71]. This rate was lower in the *E*. *muticus* genome than that of *L*. *savala*, which may be the main reason for the significantly lower genome size of the former. However, the heterozygosity rate of the *E*. *muticus* genome was 2.4 times higher than that of *L*. *savala*, indicating that *E*. *muticus* may have a relatively high level of genetic variation. Meng et al. [22] demonstrated that the randomly amplified polymorphic DNA (RAPD) polymorphism rate and genetic polymorphism were higher in *E*. *muticus* from the Yellow Sea than that of *Trichiurus lepturus*.

The number of chromosomes in the assembled genome of *E*. *muticus* was 24, consistent with that of *L*. *savala*, *T*. *albacares*, and *T*. *maccoyii* [23]. The ML tree results indicated that the phylogenetic relationships of these four species were consistent with their morphological classification [72]. The number of protein-coding genes annotated in the *E*. *muticus* genome was 21,949, which was significantly fewer than that in *L*. *savala* (23,625), *T*. *albacares* (BioProject: PRJEB47267) (24,623), and *T*. *maccoyii* (BioProject: PRJEB46021) (24,659). This may be related to their genome sizes (709.27 Mb vs. 790.02 Mb, 792.1 Mb, 782.4 Mb). Although a high level of collinearity was observed between the genomes of *E*. *muticus* and *L*. *savala*, there were varying degrees of gene deletions on chromosomes 2, 3, and 24 in the *E*. *muticus* genome compared to *L*. *savala*. This may be an important reason for the differences in biological characteristics between the two species.

### 4.2. Role of Autophagy-Related Genes in the Formation of Whip-like Tail in E. muticus

Autophagy refers to several processes by which cytoplasmic substances are introduced into the lysosome for degradation by autophagosomes [73,74]. Autophagy is involved in cell apoptosis and tissue remodeling during embryogenesis [75], responsible for the degradation of normal proteins to reorganize cells during animal metamorphosis and development [76]. It has been confirmed that autophagy is an important part of organ degeneration in arthropods and organ metamorphosis remodeling in most lepidopteran larvae [77,78]. A previous study reported that autophagosomes were present in the silk gland organ of the silkworm (*Bombyx mori*) during metamorphosis [79]. Moreover, the expression levels of both BmAtg8 and BmAtg12 proteins in the silk gland of the fifth instar larvae of *B. mori* were obviously up-regulated [80]. This indicates that autophagy plays a crucial role in the differentiation and degeneration of silk glands in the silkworm.

In the positive selection analyses, we screened several genes related to autophagosome formation (*atg5*, *atg7*, *map1lc3b*, and *map1lc3c*) and lysosome-related genes (*ids*, *lipa*, *gla*, *man2b1*, *glns*, *dnase2*, *ppt1*, and *lamp2*). These genes are crucial for the autophagy process in *E*. *muticus*, and may be involved in the autophagy-mediated degradation of certain organs. The protein encoded by *atg5* plays a core role in autophagy [81], and reducing or knocking out this protein could down-regulate or completely inhibit autophagy [82]. This interacts with the Atg12 protein to form an Atg12-Atg5 conjugate, which participates in the elongation of the isolation membranes during autophagosome formation [83]. As an essential element of autophagy, *atg7* encodes an E1-like activating enzyme involved in the two ubiquitin-like systems required for autophagy [84,85]. The absence of Atg7 protein could impair the degradation of the inner autophagosomal membrane (IAM) after the fusion of autophagosomes and lysosomes [86]. The *map1lc3b* and *map1lc3c* genes encode the Map1lc3 protein, an ortholog of the yeast autophagosome protein Atg8 [87], mediating autophagosome membrane formation as a ubiquitin-like modifier [88]. During the midgut remodeling in the larvae of *B. mori*, the expression levels of autophagy-related genes (*atg5*, *atg6*, and *atg8*) were significantly up-regulated [89]. Similarly, there was a significant increase in the expression levels of Atg5 and Lc3 proteins (orthologue of Atg8 protein) in the injury regeneration site of the caudal fin of zebrafish (*D. rerio*), indicating that *atg*-mediated autophagy is key for caudal fin regeneration in zebrafish [90]. In the *L*. *savala* genome, autophagy-related genes such as *atg3*, *atg4c*, and *atg12*, which are associated with the formation of its elongated whip-like tail, were also detected by positive selection analyses [23]. Meanwhile, the *ids*, *lipa*, *gla*, *man2b1*, *glns*, *dnase2*, and *ppt1* genes detected in this study encode various acid hydrolases that function in lysosomes. The *lamp2* gene encodes lysosomal-associated membrane protein 2, which protects lysosomal membranes from degradation by hydrolases [91]. These genes are essential for the lysosomal degradation of cellular substances and the maintenance of intracellular stability and autophagy [92]. Based on these findings, we speculate that the positive selection genes involved in autophagy may play a crucial role in the formation of the whip-like tail in *E*. *muticus*, especially the *atg5*, *atg7*, *map1lc3b*, and *map1lc3c* genes.

### 4.3. Evolution of Innate Immune System in E. muticus

Through positive selection and gene family expansion analyses, we identified several genes (*il1rl1*, *il17a*, *c1s*, *c1qa*, *c9*, *mif*, *ccr3*, and *ccr5*) related to innate immunity in the *E*. *muticus* genome. The *il1rl1* and *il17a* genes are associated with interleukins and are involved in the expression and regulation of inflammatory immune responses [93,94]. The *il1rl1* gene encodes the interleukin 1 receptor-like 1 protein (also known as St2) [95], which induces an immune response through its only ligand, interleukin-33 [96]. Conversely, *il17a* encodes the interleukin-17A protein [97] and plays an important role in the innate immune response, adaptive immunity, and immune defense against bacteria in teleost fishes [98]. The *c1s*, *c1qa*, and *c9* genes belong to the serum complement system-related genes, and *c1s* encodes a serine protease (C1s) [99], while *c1qa* encodes the A-chain polypeptide of serum complement subcomponent C1q [100]. Both C1s and C1q are components of the serum complement system C1, which defends against microbial infections and maintains immune homeostasis in organisms [101]. The complement C9 protein encoded by *c9* is critical for the innate immune response of teleost fishes against pathogen invasion [102]. The *mif* gene encodes the macrophage migration inhibitory factor [103], which enhances the resistance to bacterial invasion and promotes innate immune responses in golden pompano (*Trachinotus ovatus*) [104]. The *ccr3* and *ccr5* genes encode chemokine receptor family proteins that coordinate immune cell localization and function in the immune response of aquatic animals [105]. These genes were mainly enriched in pathways such as cytokine–cytokine receptor interaction, complement and coagulation cascades, and virus protein interaction with cytokine and cytokine receptors, indicating that they are crucial for the innate immunity of *E*. *muticus*. This may also indicate that the immune system of *E*. *muticus* has evolved. Similarly, several immune-related genes (e.g., *cfi*, *c1qa*, *vtn* genes, and the *il* gene family) detected in the *L*. *savala* genome were also significantly enriched in the complement and coagulation cascades and cytokine–cytokine receptor interaction pathways [23]. Moreover, the *c1qa* gene and the *il* gene families were identified in both *E*. *muticus* and *L*. *savala*, indicating that these two genes are conserved and are important for the immune system of trichiurids.

It has been demonstrated that pathogens, including bacteria, viruses, and parasites, could enhance evolutionary selective pressure on the host and promote the evolution of the host’s immune system [106,107]. You et al. [108] observed that several immune-domain-containing genes, including the Toll-like receptor 13 (*tlr13*) gene family (the family of innate immune receptors), were commonly present in the genomes of four representative mudskippers [109]. These genes may provide a special immune defense against novel pathogens encountered by mudskippers when living on land. In the genome of brown-marbled grouper (*E. fuscoguttatus)*, Yang et al. [110] identified several expanded gene families (such as *nlrc3*, *igl*, *trim25*, *fcrl*, and *trim35*) closely related to its antiviral infection, suggesting that the grouper has undergone adaptive evolution for disease resistance. In other words, the pathogen-rich habitats might have been the driving force behind its immune system’s evolution. Another recent transcriptome analysis found that the peanut worm (*Sipunculus nudus*) has evolved different molecular mechanisms of immune responses to various habitat environments of the intertidal zone [111]. *E*. *muticus* has a wide range of habitats across the tropical, subtropical, and temperate zones of the Indo-West Pacific Oceans, rich in bacteria and diverse viruses. As previously reported, the East China Sea shelf sediments contained at least 13 bacteria phyla [112] and had abundant viral communities in their surface waters, including at least 1029 virus species [113]. Moreover, the bacterial communities in the deep subseafloor sediments of the western Pacific warm pool contained more than five groups (e.g., α-/β-Proteobacteria) [114], and a highly diversified bacterial community (seven dominant bacterial groups) was determined in the equatorial region of the East Indian Ocean and adjacent Bay of Bengal waters, with seven dominant bacterial phyla (e.g., Proteobacteria, Bacteroidetes, Actinobacteria) [115]. However, our study could not clarify the correlation between the immune system of *E*. *muticus* and its habitat environments due to the lack of relevant research data on its pathogens and diseases. Nonetheless, the innate immune evolution of *E*. *muticus* detected in this study provides a natural barrier against the invasion of several pathogens in its vast habitats, which may be important for maintaining the widespread distribution and population stability of *E*. *muticus*.

### 4.4. Important Role of DNA Repair-Related Genes in Maintaining Genome Stability of E. muticus

Genome integrity and stability are prerequisites for the survival and reproduction of species [116]. The genome of a species is constantly affected by internal and external factors during its life history, leading to DNA damage (e.g., DNA replication errors, ultraviolet radiation, environmental or reagent contamination) [117]. Therefore, repairing damaged DNA is important for maintaining the stability of a species’ genome [118]. The Fanconi anemia pathway is essential for repairing damaged DNA and is primarily used to repair the interstrand DNA cross-linking damage [119]. Interstrand DNA cross-linking is a common type of DNA damage caused by ultraviolet light (UV), aldehydes produced by cellular metabolism, and exogenous DNA cross-linking agents, which block DNA replication, transcription, and recombination [118,120]. In our positive selection analyses, the genes (*fanca*, *fance*, *fanci*, *faap100*, *eme1*, *brip1*, *blm*, *slx1a*, *polh*, *palb2*, and *brca1*) related to DNA repair in *E*. *muticus* were significantly enriched in the Fanconi anemia pathway. The proteins encoded by *fanca*, *fance*, *fanci*, and *faap100* are components of the Fanconi anemia core complex, which are essential for DNA repair and maintenance of genomic stability [121,122]. Fanca protein activates interstrand DNA cross-link repair by monoubiquitination of Fancd2 [123]. Fance protein forms a ternary complex with Fancc and Fancd2 proteins and plays a role in the DNA damage response [124]. Fanci protein is critical for the repair of DNA double-strand breaks and interstrand DNA cross-links [125,126]. Faap100 protein regulates Fancd2 monoubiquitination and the stability of the Fanconi anemia core complex, which could significantly affect the DNA damage response associated with Fanconi anemia [127]. Furthermore, the proteins encoded by *eme1*, *brip1*, *blm*, *slx1a*, and *polh* are important enzymes involved in the DNA repair process. Among them, the Eme1 and Mus81 proteins form a DNA endonuclease that cleaves the branching DNA structures [128], and the lack of Eme1 would lead to chromosomal instability in mouse clonal cells [129]. The *brip1* gene encodes a 5′ to 3′ DNA helicase (acting in DNA double-strand break repair) required to maintain chromosomal stability [130]. Like helicase encoded by *blm*, Blm RecQ participates in DNA replication and repair [131,132]. The *palb2* gene encodes the partner and localizer of Brca2, which plays a key role in the homologous recombination repair by localizing to DNA damage sites [133]. As a functional unit component of the homologous recombination and DNA damage repair [134], the Brca1 protein encoded by *brca1* participates in DNA damage repair and transcriptional regulation [135,136]. Thus, it is evident that the proteins encoded by these genes are important components of the DNA repair system of *E*. *muticus*.

Similarly, genes related to DNA repair (*polm*, *prkdc*, *bard1*, *brca1*, *nbn*, *xrcc2*, *eme2*, and *faap100*) were also screened in the positive selection analyses of the *L*. *savala* genome [23]. These genes have an essential contribution to the recombination of homologous chromosomes and the maintenance of genomic stability in the *L*. *savala* genome [23]. Based on a comparative analysis of the genomes of chondrichthyan and teleost fishes, Marra et al. [137] found that most positively selected genes associated with DNA damage response, DNA repair, translesion DNA synthesis, and ubiquitination widely exist in chondrichthyan fishes (e.g., white shark (*Carcharodon carcharias*), whale shark (*R. typus*), and elephant shark (*Callorhinchus milii*)), and are important in maintaining the genomic stability of sharks. Further positive selection analyses showed that some core histone genes were involved in the DNA damage response and associated histone epigenetic modifications in white shark, and that genes (*fgg*, *extl2*, and *krt18*) and terms (e.g., angiogenesis, VEGFA-VEGFR2 signaling network, epidermal growth factor receptor) related to the stronger wound healing in white shark were also identified [137]. This revealed the pivotal role of the white shark’s cancer-fighting, long lifespan, and superior wound-healing ability in maintaining its genome stability and conservation genes, which perpetuates the long-term existence of the species [137]. In a genomic analysis of the Yap hadal snailfish, 34 positively selected genes (e.g., *rad52*, *rad9a*, *ercc1*, *exo1*, *pms1*, and *polk*) were identified to be significantly enriched in the DNA repair pathway, and its copies of *rad51* and *xrcc2* were higher than those of other teleost fishes [138]. Two proteins encoded by *rad51* and *xrcc2* play key roles in repairing DNA double-strand breaks [139] and DNA damage [140], respectively. Recently, 22 significantly co-expanded gene families of the two deep-sea anemones (*Alvinactis idsseensis* and *Paraphelliactis xishaensis*) were found to be associated with DNA repair and cell membrane [141]. High hydrostatic pressure can cause DNA breaks and damage and affect cell membrane fluidity, protein stability, and the cytoskeleton [141]. Thus, the identification of these genes signifies that the Yap hadal snailfish and deep-sea anemones have a greater ability to repair DNA, which is critical for their adaptation to the high hydrostatic pressure in their living environment [138]. Based on these analyses, we infer that the genes related to DNA repair in *E*. *muticus* may have important contributions in maintaining its genome stability and survival. Although no studies have been reported on the DNA repair mechanisms in trichiurids, we screened multiple genes related to DNA repair in the genomes of both *E*. *muticus* and *L*. *savala*, indicating that the trichiurids may have well-developed DNA repair mechanisms. However, the importance of these genes in these species remains to be explored.

## 5. Conclusions

In this study, a high-quality *E*. *muticus* genome was assembled at the chromosomal level for the first time via PacBio sequencing and Hi-C technology. The assembled genome size was 709.27 Mb, with a contig N50 value of 25.07 Mb, and contained 21,949 protein-coding genes and 24 chromosomes. Positive selection analyses revealed that the genes (*atg5*, *atg7*, *map1lc3b*, and *map1lc3c*) related to autophagosome formation may be the key factors associated with the whip-like tail of *E*. *muticus*. The rapid evolution of the innate immune-related genes (*il1rl1*, *il17a*, *c1s*, *c1qa*, *c9*, *mif*, *ccr3*, and *ccr5*) was detected in *E*. *muticus*. This innate immune evolution provides a natural barrier for *E*. *muticus* against the invasion of various pathogens in its vast habitats. Moreover, the genes (*fanca*, *fance*, *fanci*, *faap100*, *eme1*, *brip1*, *blm*, *slx1a*, *polh*, *palb2*, and *brca1*) related to DNA repair mechanisms were also identified in *E*. *muticus*. These genes are important in maintaining the stability of the *E*. *muticus* genome and ensuring the survival and reproduction of the species. Thus, our study provides important basic data for exploring the genetic and evolutionary mechanisms of *E*. *muticus* at the genomic level and is an invaluable reference for the genomic studies of other trichiurids.

## Figures and Tables

**Table 1:** Statistics of the sequencing data of the *E*. *muticus* genome.

Type	Platform	Library Size (bp)	Raw Data (Gb)	Clean Data (Gb)	Coverage (×)
Illumina Nova	Illumina NovaSeq-6000	350	71.40	63.03	99.1
PacBio SMRT	PacBio Sequel II	15 k	59.57	34.97	48.5
Hi-C	Illumina NovaSeq-6000	350	75.20	74.25	104.4
Illumina RNA-Seq	Illumina NovaSeq-6000	350	7.10	6.71	9.8
ONT RNA-Seq	NanoPromethION	-	9.09	8.61	12.6
Total			222.36	187.57	274.4

**Table 2:** Gene structure and parameters predicted by three methods.

Methods	Gene Set	Number	Average Gene Length (bp)	Average CDS Length (bp)	Average Exon Per Gene	Average Exon Length (bp)	Average Intron Length (bp)
De novo	Genscan	36,114	13,128.07	1419.77	7.78	182.56	1727.59
	AUGUSTUS	36,217	9868.25	1270.82	6.96	182.72	1443.6
Homolog	*Etheostoma spectabile*	30,817	12,295.23	1472.87	8.29	177.59	1483.41
	*Homo sapiens*	24,338	13,205.29	1321.50	8.24	160.32	1640.46
	*Larimichthys crocea*	32,085	12,203.04	1478.14	8.27	178.64	1474.12
	*Thunnus maccoyii*	32,571	12,375.79	1501.14	8.32	180.47	1485.64
	*Perca flavescens*	31,479	12,242.79	1463.95	8.30	176.36	1475.98
	*Takifugu rubripes*	28,393	12,451.37	1509.68	8.63	174.96	1433.9
	*Thunnus albacares*	32,146	12,393.48	1508.74	8.43	179.07	1465.57
	*Danio rerio*	29,225	12,428.59	1442.84	8.26	174.65	1512.68
	*Sander lucioperca*	32,372	12,404.23	1482.49	8.32	178.23	1492.16
	*Oryzias latipes*	30,041	12,304.19	1507.13	8.34	180.76	1471.05
Transcriptome	RNAseq	10,859	17,854.09	1764.30	11.53	312.82	1353.38
	ISOseq	1698	10,846.91	1173.28	9.55	231.66	1010.05
BUSCO		3661	11,770.51	1843.36	12.31	149.74	877.63
MAKER		22,903	14,826.32	1668.48	9.72	259.16	1411.85
HiCESAP		21,949	15,159.85	1727.52	10.27	259.53	1347.92

**Table 3:** The gene family clustering in 20 fish species.

Species	Genes	Unclustered	Genes	Family	Unique	Unique	Common	Common	Single-Copy
	Number	Genes	In Families	Number	Families	Families Genes	Families	Families Genes	Genes
*Eupleurogrammus muticus*	21,949	1123	20,826	15,686	25	89	6711	10,925	3006
*Lepturacanthus savala*	23,625	2040	21,585	15,681	47	926	6711	10,880	3006
*Thunnus albacares*	24,623	429	24,194	17,161	23	49	6711	11,427	3006
*Thunnus maccoyii*	24,646	475	24,171	17,180	22	61	6711	11,423	3006
*Hippocampus comes*	21,175	1439	19,736	14,602	67	170	6711	10,931	3006
*Acanthopagrus latus*	23,773	405	23,368	16,666	57	168	6711	11,429	3006
*Cheilinus undulatus*	23,303	521	22,782	15,995	86	410	6711	11,358	3006
*Echeneis naucrates*	21,275	194	21,081	15,518	21	62	6711	11,220	3006
*Epinephelus akaara*	23,923	1322	22,601	16,077	66	184	6711	11,659	3006
*Epinephelus fuscoguttatus*	24,005	1055	22,950	16,191	121	338	6711	11,574	3006
*Larimichthys crocea*	23,354	660	22,694	16,573	43	118	6711	11,372	3006
*Latimeria chalumnae*	20,932	3250	17,682	13,208	174	696	6711	9681	3006
*Monopterus albus*	21,343	915	20,428	15,304	41	99	6711	10,979	3006
*Perca flavescens*	23,736	739	22,997	16,417	41	122	6711	11,376	3006
*Periophthalmus magnuspinnatus*	21,293	597	20,696	15,033	53	223	6711	11,140	3006
*Rhincodon typus*	21,868	4491	17,377	12,554	193	662	6711	10,029	3006
*Sander lucioperca*	24,714	687	24,027	16,892	60	157	6711	11,484	3006
*Takifugu flavidus*	29,408	4177	25,231	15,690	261	1349	6711	10,980	3006
*Takifugu rubripes*	22,064	407	21,657	15,320	42	107	6711	11,445	3006
*Tetraodon nigroviridis*	27,918	7741	20,177	14,512	222	541	6711	11,285	3006

**Table 4:** KEGG enrichment analysis of expanded and contracted gene families.

1. Expansion (67 Gene Families, Top 20 KEGG Pathways, *p*-Value < 0.05)		
KEGG Pathways	*p*-Value	Genes
Ascorbate and aldarate metabolism	2.97 × 10^−7^	*ugt3*, *ugt1a1*
Pentose and glucuronate interconversions	5.85 × 10^−7^	*ugt3*, *ugt1a1*
Chemical carcinogenesis—DNA adducts	5.85 × 10^−7^	*ugt3*, *ugt1a1*
Porphyrin and chlorophyll metabolism	7.94 × 10^−7^	*ugt3*, *ugt1a1*
Drug metabolism—cytochrome P450	1.21 × 10^−6^	*ugt3*, *ugt1a1*
Metabolism of xenobiotics by cytochrome P450	1.39 × 10^−6^	*ugt3*, *ugt1a1*
Notch signaling pathway	1.97 × 10^−6^	*hes5*
Steroid hormone biosynthesis	3.58 × 10^−6^	*ugt3*, *ugt1a1*
Retinol metabolism	8.61 × 10^−6^	*ugt3*, *ugt1a1*
Drug metabolism—other enzymes	2.24 × 10^−5^	*ugt3*, *ugt1a1*
Steroid biosynthesis	1.74 × 10^−4^	*soat1*
Bile secretion	2.24 × 10^−4^	*ugt3*, *ugt1a1*
Viral protein interaction with cytokine and cytokine receptor	5.96 × 10^−4^	*ccr3*, *ccr5*
Breast cancer	8.91 × 10^−4^	*hes5*
Hypertrophic cardiomyopathy	0.001042306	*ttn*
Chemokine signaling pathway	0.001245404	*tiam1*, *ccr3*, *ccr5*
Epithelial cell signaling in Helicobacter pylori infection	0.001703999	*ptprz1*
Human papillomavirus infection	0.001826957	*hes5*, *dlg1l*
Dilated cardiomyopathy	0.002127476	*ttn*
Cholesterol metabolism	0.004796358	*soat1*
**2. Contraction (123 gene families, Top 20 KEGG pathways, *p*-value < 0.05)**		
**KEGG Pathways**	***p*-Value**	**Genes**
ABC transporters	4.31 × 10^−20^	*abcc8*, *abcc12*, *abcc10*, *abcc5*, etc.
Axon guidance	2.48 × 10^−19^	*epha2*, *epha8*, *epha3*, *epha6*, etc.
Antifolate resistance	4.78 × 10^−11^	*abcc4*, *abcc5*, *abcc2*, *abcc3*, etc.
Antigen processing and presentation	2.85 × 10^−9^	*hspa5*, *hsc71*, *hspa8*, *hsc70*
Legionellosis	1.63 × 10^−7^	*hsc71*, *hspa8*, *hsp70*
Longevity regulating pathway—multiple species	8.54 × 10^−7^	*hsc71*, *hspa8*, *hsp70*
Systemic lupus erythematosus	2.52 × 10^−6^	*h3f3b*, *hist2h3d*
Protein processing in endoplasmic reticulum	2.71 × 10^−6^	*hsc71*, *hspa8*, *hspa5*, *hsp70*
Toxoplasmosis	5.46 × 10^−6^	*hsc71*, *hspa8*, *hsp70*
Spliceosome	5.70 × 10^−6^	*hsc71*, *hspa8*, *hsp70*
Measles	1.04 × 10^−5^	*hsc71*, *hspa8*, *hsp70*
MAPK signaling pathway	1.06 × 10^−5^	*hsc71*, *hspa8*, *epha2*, *hsp70*
Estrogen signaling pathway	1.89 × 10^−5^	*hsc71*, *hspa8*, *hsp70*
Lipid and atherosclerosis	2.53 × 10^−5^	*hspa5*, *hsc71*, *hspa8*, *hsp70*
Neutrophil extracellular trap formation	3.25 × 10^−5^	*h3f3b*, *hist2h3d*
Alcoholism	4.49 × 10^−5^	*h3f3b*, *hist2h3d*
Prion disease	1.26 × 10^−4^	*hsc71*, *hspa5*, *hspa8*, *hsp70*
Transcriptional misregulation in cancer	2.07 × 10^−4^	*h3f3b*, *hist2h3d*
Vitamin digestion and absorption	2.46 × 10^−4^	*abcc1*
Bile secretion	4.76 × 10^−4^	*abcc4*, *abcc2*, *abcc3*

**Table 5:** The results of the positive selection analysis of *E*. *muticus*.

Group 1 (Genes: 1566; KEGG Pathways: 21, *p*-Value < 0.05)		
(*Eupleurogrammus muticus* and *Lepturacanthus savala*) vs. (*Acanthopagrus latus*, *Epinephelus fuscoguttatus*, *Epinephelus akaara*, *Perca flavescens*, *Larimichthys crocea*, and *Sander lucioperca*)		
KEGG Pathways	*p*-Value	Genes
Novobiocin biosynthesis	0.00	*tat*
Fanconi anemia pathway	3.59 × 10^−6^	*slx1a*, *eme1*, *palb2*, *fanca*, etc.
Non-homologous end-joining	0.001790458	*polm*, *nhej1*, *dntt*
Homologous recombination	0.003095449	*eme1*, *blm*, *brca1*, *bard1*, etc.
Ferroptosis	0.004991427	*atg5*, *atg7*, *map1lc3b*, *map1lc3c*, etc.
Bacterial secretion system	0.004708158	*srp54*
Base excision repair	0.006208329	*mpg*, *parp4*, *smug1*, *neil3*, etc.
RNA transport	0.007638419	*eif2b3*, *gemin5*, *nup188*, *acin1*, etc.
Oxidative phosphorylation	0.009939509	*ndufb9*, *ppa1*, *atp6v0b*, *atp5g3*, etc.
Cell cycle—caulobacter	0.01347929	*clpp*
Sesquiterpenoid and triterpenoid biosynthesis	0.01347929	*sqle*
Caffeine metabolism	0.02573447	*uox*
Aminobenzoate degradation	0.02573447	*echs1*
Naphthalene degradation	0.02573447	*adh5*
DNA replication	0.03018216	*rnaseh2b*, *zmcm3*, *dna2*, *rfc1*, etc.
Autophagy—yeast	0.03827173	*rab7*, *ip6k1*, *vps8*, *kras*, etc.
Ribosome biogenesis in eukaryotes	0.03438198	*eif6*, *nob1*, *pop4*, *dkc1*, etc.
One carbon pool by folate	0.03736458	*dhfr*, *mthfr*, *mthfd2*, etc.
Cytokine–cytokine receptor interaction	0.04263281	*il1rl1*, *il17a*, *ngfr*, *tnfrsf1b*, etc.
Ubiquinone and other terpenoid-quinone biosynthesis	0.04447989	*tat*, *coq6*
Citrate cycle (TCA cycle)	0.04820553	*dld*, *dlat*, *sdha*, *suclg1*, etc.
**Group 2 (Genes: 1022; KEGG Pathways: 20, *p*-Value < 0.05)**		
**(*Eupleurogrammus muticus*) vs. (*Acanthopagrus latus*, *Epinephelus fuscoguttatus*, *Epinephelus akaara*, *Perca flavescens*, *Larimichthys crocea*, *and Sander lucioperca*)**		
**KEGG Pathways**	***p*-Value**	**Genes**
Novobiocin biosynthesis	0.00	*tat*
Fanconi anemia pathway	7.11 × 10^−8^	*palb2*, *fance*, *faap100*, *blm*, etc.
Homologous recombination	0.000144	*eme1*, *xrcc3*, *palb2*, *brip1*, etc.
Mismatch repair	0.00209	*msh3*, *pold1*, *exo1*
Cytokine–cytokine receptor interaction	0.00485	*il1rl1*, *ngfr*, *prlr*, *il22ra1*, etc.
Aminobenzoate degradation	0.0105	*ehhadh*
Lysosome	0.012	*lamp2*, *lipa*, *man2b1*, *ppt1*, etc.
Base excision repair	0.0127	*mpg*, *parp4*, *pold1*
Notch signaling pathway	0.0151	*cir1*, *dtx3*, *dtx2*, *maml2*, etc.
Glycosylphosphatidylinositol (GPI)-anchor biosynthesis	0.0157	*pigk*, *pigo*, *pgap1*
Ribosome biogenesis in eukaryotes	0.0163	*drosha*, *riok2*, *dkc1*, *rpp40*, etc.
Non-homologous end-joining	0.0203	*nhej1*
Tropane, piperidine and pyridine alkaloid biosynthesis	0.0337	*tat*
Caprolactam degradation	0.0337	*ehhadh*
JAK-STAT signaling pathway	0.0358	*ccnd3*, *il22ra1*, *prlr*, *il12b*, etc.
Ribosome	0.0361	*rps26*, *rpl29*, *mrps18c*, *rpl23*, etc.
Aminoacyl-tRNA biosynthesis	0.0397	*sars*, *sepsecs*, *wars2*
Nucleotide excision repair	0.0432	*ercc3*, *ercc5*, *pold1*, *ccnh*
Phenylalanine, tyrosine and tryptophan biosynthesis	0.0437	*tat*
Phenylalanine metabolism	0.0461	*tat*, *mif*
**Group 3 (Genes: 2300; KEGG Pathways: 17, *p*-Value < 0.05)**		
**(*Eupleurogrammus muticus and Lepturacanthus savala*) vs. (*Thunnus albacares* and *Thunnus maccoyii*)**		
**KEGG Pathways**	***p*-Value**	**Genes**
Cytokine–cytokine receptor interaction	5.95 × 10^−5^	*ngfr*, *ccr6*, *il17a*, *il22ra1*, etc.
Fanconi anemia pathway	6.21 × 10^−4^	*eme1*, *palb2*, *fanca*, *brip1*, etc.
Base excision repair	7.34 × 10^−4^	*mpg*, *lig3*, *smug1*, *nthl1*, etc.
Viral protein interaction with cytokine and cytokine receptor	8.07 × 10^−4^	*il6*, *ccr6*, *tnfsf14*, *il22ra1*, etc.
Lipoic acid metabolism	0.009138648	*lipt2*
Sphingolipid metabolism	0.01278764	*psap*, *plpp3*, *cerk*, *cers5*, etc.
DNA replication	0.01548808	*rnaseh2b*, *pole2*, *dna2*, *prim2*, etc.
Glycosylphosphatidylinositol (GPI)-anchor biosynthesis	0.01839599	*pigk*, *pigo*, *pigw*, *pigt*, etc.
Complement and coagulation cascades	0.01912686	*c5*, *c9*, *c1s*, *c1qa*, etc.
Ribosome	0.02090745	*mrpl16*, *rps10*, *mrpl21*, *mrpl11*, etc.
Ubiquinone and other terpenoid-quinone biosynthesis	0.02202827	*tat*, *vkorc1l1*, *coq6*
Nucleotide excision repair	0.02460608	*rbx1*, *gtf2h3*, *pole2*, *ccnh*, etc.
Biotin metabolism	0.02567046	*hlcs*
RNA transport	0.03514058	*eif2b3*, *nup188*, *gemin5*, *gemin6*, etc.
JAK-STAT signaling pathway	0.03880553	*prl*, *il6*, *lifr*, *csf2rb*, etc.
Caffeine metabolism	0.04809981	*uox*
Apoptosis—multiple species	0.04856226	*ngfr*, *cyc*, *tnfrsfla*, *diablo*, etc.

## Data Availability

The genome assembly data of *Eupleurogrammus muticus* were deposited at NCBI under BioProject ID: PRJNA1040966 (Submitted), BioSample accession: SAMN38271036 (Submitted). The raw read sequence accession numbers: SRR26857548, SRR26857549, SRR26857550, SRR26857546, SRR26857547.

## Supplementary Materials

The following supporting information can be downloaded at https://www.mdpi.com/article/10.3390/ani14030434/s1. Supplementary Material S1: Figure S1: The K-mer analysis of *E. muticus* genome; Figure S2: The correlation graph between the GC content and average depth distribution based on Illumina clean reads (A) and PicBio HiFi reads (B); Figure S3: The number of reads in the valid match result; Figure S4: Statistical graphs of the gene set obtained via gene structure prediction (comparison graph of gene elements with closely related species); Figure S5: The phylogenetic tree of 20 fish species; Figure S6: Whole-genome sequence-based collinearity analysis of *E. muticus* and *L. savala*; Table S1: Blast results of the NCBI nucleotide database; Table S2: Sequence statistics of genome assembly results; Table S3: The results of the BUSCO evaluation; Table S4: The statistics of matching results; Table S5: Filtering statistics of the successfully paired matches; Table S6: The results of the Hi-C-assisted assembly; Table S7: The interspersed repeat classification results; Table S8: Annotation and detailed data of the non-coding RNA; Table S9: Summary of functional annotations of the predicted genes. Supplementary Material S2: Results of enrichment analysis of unique gene families in *E*. *muticus*. Supplementary Material S3: Results of enrichment analysis of the contraction and expansion gene families in *E*. *muticus*. Supplementary Material S4: Results of the positive selection analysis of *E*. *muticus*.
