# Supplementary material for: Whole-Genome Sequencing Analyses Reveal the Whip-like Tail Formation, Innate Immune Evolution, and DNA Repair Mechanisms of Eupleurogrammus muticus
Source: Animals (Basel). 2024 Jan 29;14(3):434. doi: 10.3390/ani14030434 (PMC10854985; doi:10.3390/ani14030434)
Supplement: Supplementary file 1 [file animals-14-00434-s001.zip › Supplementary material S1.pdf]

Supplementary Materials 1

Table S1. Blast results of the NCBI nucleotide database.

Species	Blast number	Total blast number	Total (%)
Epinephelus	369	3,850	9.58
Lateolabrax	317	3,850	8.23
Plectropomus	253	3,850	6.57
Trachurus	184	3,850	4.78
Sparus	175	3,850	4.55
Larimichthys	175	3,850	4.55
Nibea	161	3,850	4.18
Sphaeramia	139	3,850	3.61
Cottoperca	130	3,850	3.38
Neostethus	119	3,850	3.09
Myripristis	106	3,850	2.75
Acanthopagrus	87	3,850	2.26
Echeneis	84	3,850	2.18
Parambassis	84	3,850	2.18
Mastacembelus	65	3,850	1.69
Scophthalmus	64	3,850	1.66
Salarias	60	3,850	1.56
Anabas	60	3,850	1.56
Pseudochaenichthys	54	3,850	1.4
Betta	53	3,850	1.38
Oryzias	51	3,850	1.32
Trichiurus	51	3,850	1.32
Sebastes	42	3,850	1.09
Danio	41	3,850	1.06
Lates	39	3,850	1.01
Morone	36	3,850	0.94
Etheostoma	35	3,850	0.91
Seriola	34	3,850	0.88
Thalassophryne	32	3,850	0.83

Table S2. Sequence statistics of genome assembly results.

Level	Contig Length(bp)	Contig Number	Scaffold Length(bp)	Scaffold Number
N90	4,252,074	30	4,252,074	30
N80	17,884,770	22	17,884,770	22
N70	19,499,489	18	19,499,489	18
N60	23,260,739	15	23,260,739	15
N50	25,347,879	12	25,347,879	12
Total length	709,267,825	-	709,267,825	-
Max length	50,599,264	-	50,599,264	-

Table S3. The results of the BUSCO evaluation.

Type	Proteins	Percentage (%)
Complete BUSCOs	3,534	97.1
Complete and single-copy BUSCOs	3,471	95.4
Complete and duplicated BUSCOs	63	1.7
Fragmented BUSCOs	12	0.3
Missing BUSCOs	94	2.6
Total BUSCO groups searched	3,640	100

Table S4. The statistics of matching results.

	Read 1	Read 2
Unique Alignments	162,896,961	184,832,171
Multiple Alignments	33,244,412	35,772,620
Failed To Align	35,143,516	26,663,589
Paired	132,890,440	132,890,440

Table S5. Filtering statistics of the successfully paired matches.

Di-Tag Type	Di-Tag Count	Percent in Paired(%)	Percent in Total Reads(%)
Same Circularised	121,708	0.09	0.05
Same Fragment Dangling Ends	7,457,791	5.61	3.01
Same Fragment Internal	23,156,962	17.43	9.35
Re-ligation	8,306,456	6.25	3.35
Contiguous Sequence	0	0.00	0.00
Wrong Size	0	0.00	0.00
Invalid Pairs	39,042,917	29.38	15.76
Valid Pairs	93,847,523	70.62	37.89
Valid Pairs (de-duplication)	68,084,802	51.23	27.49

Table S6. The results of the Hi-C assisted assembly.

Type	Sequence length (bp)	Sequence number	Contig N50 (bp)	Scaffold N50 (bp)
Raw	709,267,825	156	25,347,879	25,347,879
Hi-C	702,496,981	84	25,078,085	30,064,390
Hi-C chr	691,679,068	24	25,078,085	30,064,390
Hi-C nochr	10,817,913	60	260,252	338,375

Table S7. The interspersed repeat classification results.

Type	RepeatMasker Length (bp)	Percent	RepeatProteinMask Length (bp)	Percent	De novo Length (bp)	Percent	Combined Length (bp)	Percent

DNA	55,801,163	7.94	7,245,044	1.03	64,944,234	9.24	102,347,937	14.57
LINE	26,800,614	3.82	13,753,279	1.96	19,974,971	2.84	36,949,851	5.26
SINE	6,662,391	0.95	0	0.00	2,930,370	0.42	9,190,875	1.31
LTR	16,942,356	2.41	7,110,372	1.01	13,647,602	1.94	25,110,984	3.57
Satellite	2,312,679	0.33	0	0.00	4,834,639	0.69	7,045,164	1.00
Simple repeat	0	0.00	0	0.00	373,529	0.05	373,529	0.05
Other	3,946	0.00	168	0.00	0	0.00	4,114	0.00
Unknown	628,365	0.09	6,999	0.00	59,812,968	8.51	60,414,541	8.60
Total	98,000,712	13.95	28,099,946	4.00	156,981,954	22.35	216,702,465	30.85

Table S8. Annotation and detailed data of the non-coding RNA.

**Type**		**Copy**	**Average length(bp)**	**Total length(bp)**	**Percent (%)**
miRNA		879	86	75,630	0.010766
tRNA		12,792	76	968,630	0.137884
rRNA	rRNA	13,817	144	1,993,777	0.283813
	18S	214	1,819	389,281	0.055414
	28S	0	0	0	0.000000
	5.8S	211	154	32,485	0.004624
	5S	13,392	117	1,572,011	0.223775
snRNA	snRNA	1,537	154	236,834	0.033713
	CD-box	210	146	30,564	0.004351
	HACA-box	75	147	11,044	0.001572
	splicing	1,242	155	192,959	0.027468
	scaRNA	10	227	2,267	0.000323

Table S9. Summary of functional annotations of the predicted genes.

	**Number**	**Percent (%)**
**Total**	21,949	
**Annotated**	21,492	97.92
**InterPro**	19,940	90.85
**GO**	15,253	69.49
**KEGG_ALL**	21,322	97.14
**KEGG_KO**	14,159	64.51
**Swissprot**	19,425	88.50
**TrEMBL**	21,392	97.46
**TF**	3,424	15.60
**Pfam**	19,218	87.56
**NR**	21,446	97.71
**KOG**	18,035	82.17
**Unannotated**	457	2.08

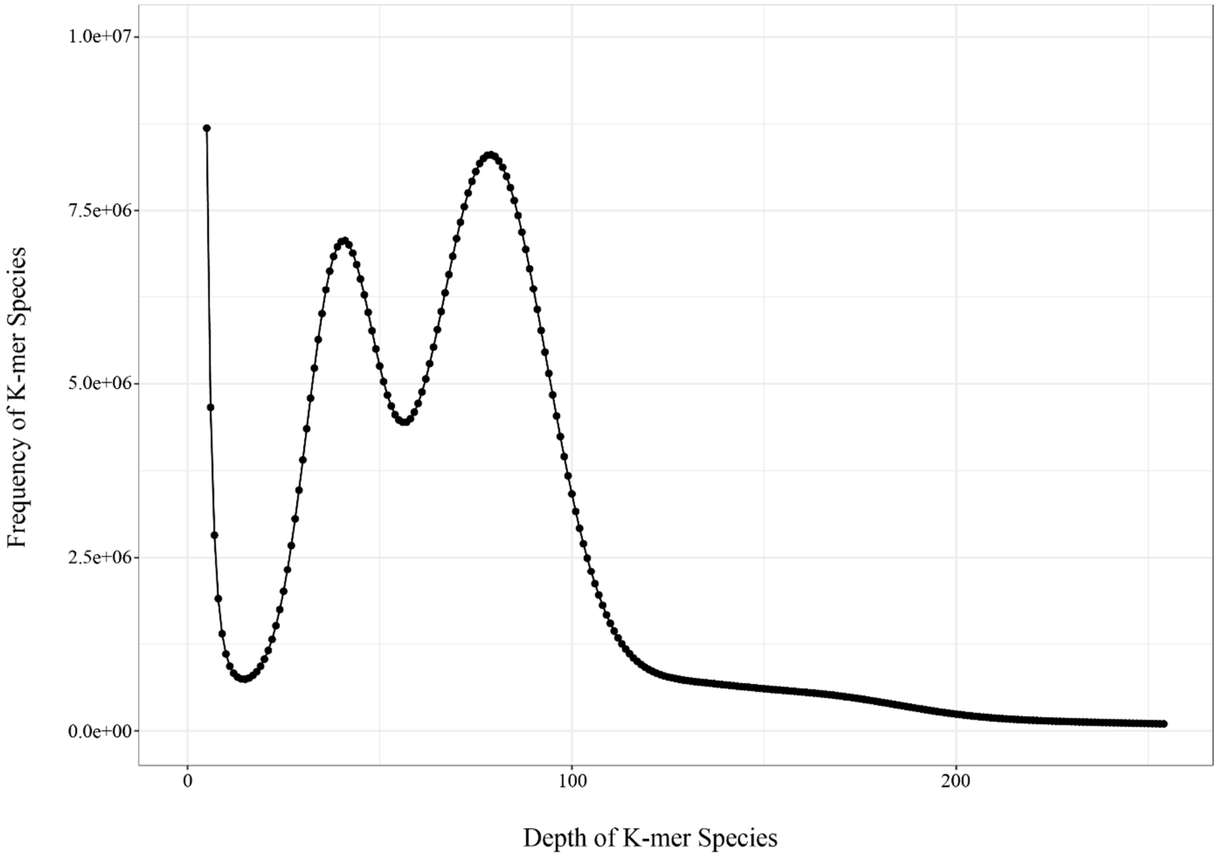

Figure S1. The K-mer analysis of *E. muticus* genome.

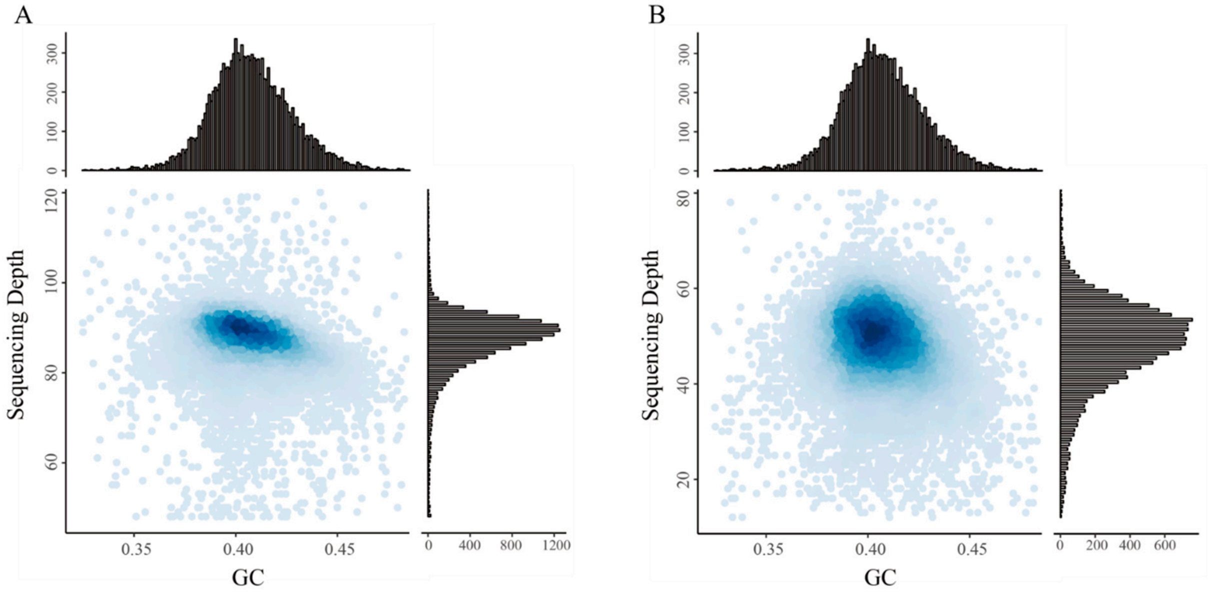

Figure S2. The correlation graph between the GC content and average depth distribution based on Illumina clean reads (A) and PicBio HiFi reads (B).

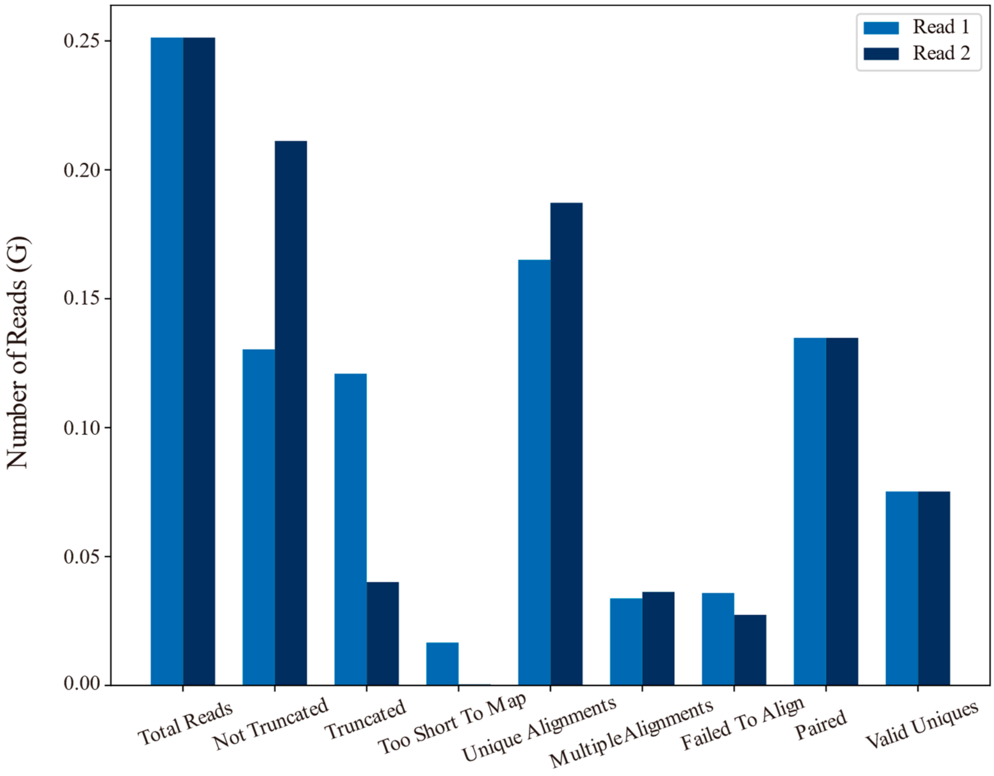

Figure S3. The number of reads in the valid match result.

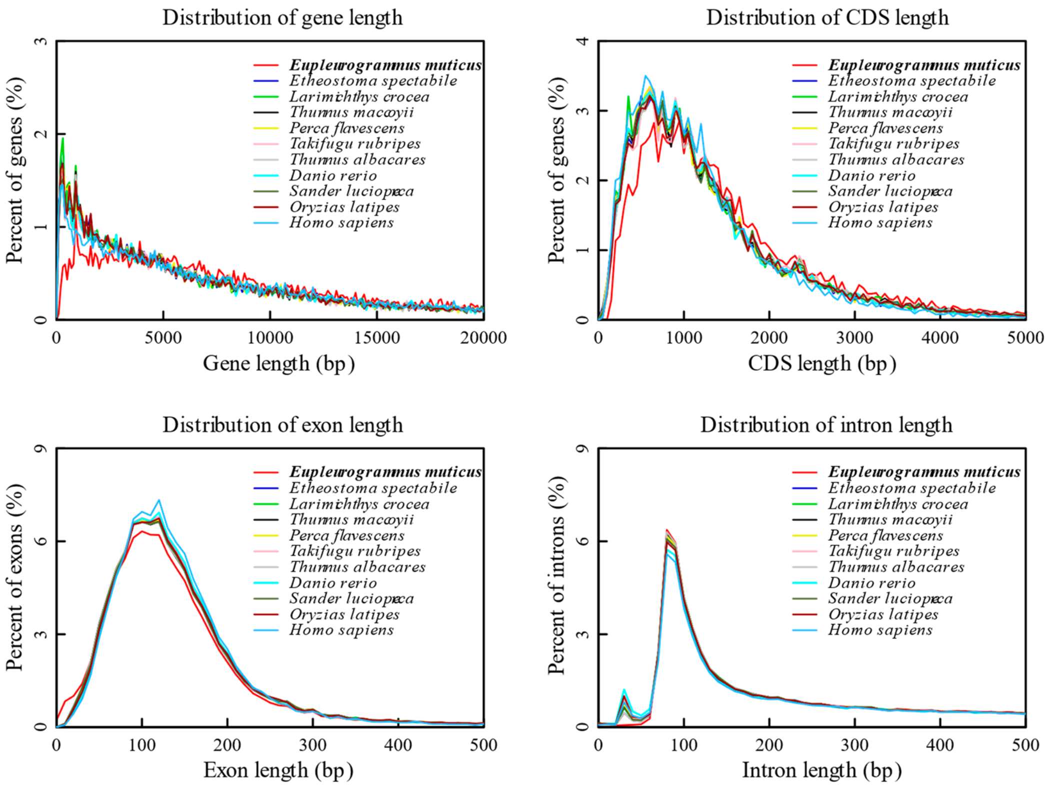

Figure S4. Statistical graphs of the gene set obtained via gene structure prediction (comparison graph of gene elements with closely related species).

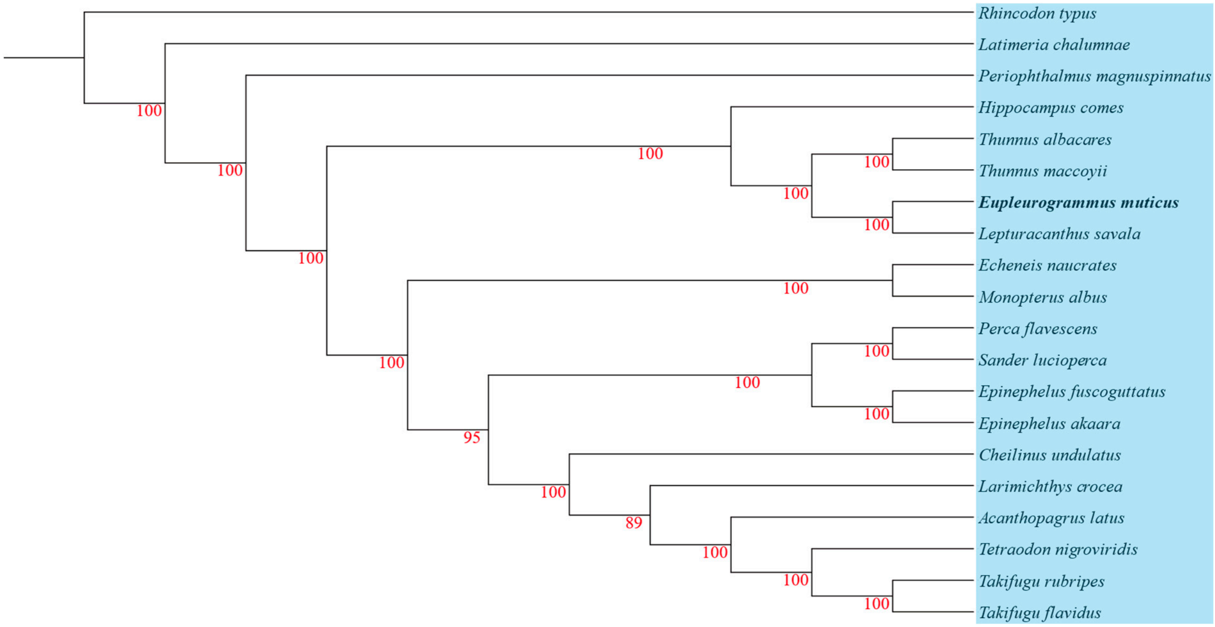

Figure S5. The phylogenetic tree of 20 fish species.

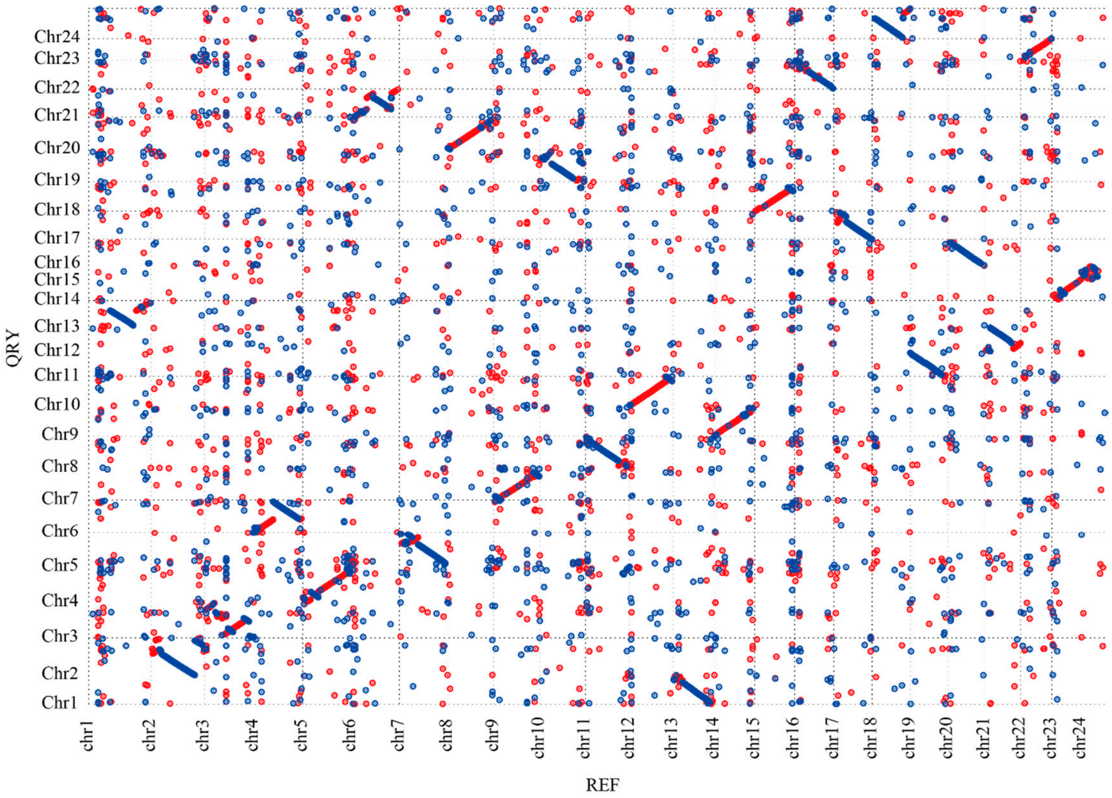

Figure S6. Whole-genome sequence-based collinearity analysis of *E. muticus* and *L. savala*.
